# Monoclonal Gammopathies of Renal Significance: Renal Biopsy and Beyond

**DOI:** 10.3390/cancers12071741

**Published:** 2020-06-30

**Authors:** Paolo Menè, Lorenzo De Alexandris, Alessandra Moioli, Salvatore Raffa, Antonella Stoppacciaro

**Affiliations:** 1 Division of Nephrology, Sant’Andrea University Hospital, Via di Grottarossa 1035–1039, 00189 Rome, Italy; 2 Divisions of Nephrology, General Pathology, and Pathology, Department of Clinical and Molecular Medicine, “Sapienza” University of Rome, 00189 Rome, Italy; lorenzo.dealexandris@uniroma1.it (L.D.A.); moioli.am@gmail.com (A.M.); salvatore.raffa@uniroma1.it (S.R.); antonella.stoppacciaro@uniroma1.it (A.S.)

**Keywords:** monoclonal gammopathies, myeloma, immunoglobulins, light chains, amyloidosis, kidney, renal biopsy

## Abstract

Monoclonal Gammopathies of Renal Significance (MGRS) are a rather heterogeneous group of renal disorders caused by a circulating monoclonal (MC) immunoglobulin (Ig) component, often in the absence of multiple myeloma (MM) or another clinically relevant lymphoproliferative disorder. Nevertheless, substantial kidney damage could occur, despite the “benign” features of the bone-marrow biopsy. One example is renal amyloidosis, often linked to a small clone of plasma cells, without the invasive features of MM. However, patients with amyloidosis may present with a nephrotic syndrome and renal failure, eventually leading to end-stage kidney disease. At the same time, other organs, such as the heart and the liver, may be severely damaged by Ig light chains or amyloid deposits, occasionally resulting in fatal arrhythmias and/or organ failure. Acute kidney injury (AKI) may as well result from massive excretion of MC proteins, with deposition disease in glomeruli or renal tubules, not rarely obstructed by luminal aggregates, or “casts”. Proliferative glomerulonephritis with monoclonal Ig deposits is another, less frequent clinical presentation of an MGRS. The present review deals with the implications of MGRS for renal function and prognosis, and the potential of tools, such as the renal biopsy, for assessing clinical risk and guiding therapy of the underlying condition.

## 1. Introduction

Monoclonal Gammopathies of Undetermined Significance (MGUS) are frequently recognized through the unexpected finding of an electrophoretically distinct monoclonal β or γ globulin peak in serum [1,2]. Individuals with such “paraprotein” usually have no evidence of a systemic hematological disease, nor organ damage, such as heart failure, liver dysfunction, bone/skeletal alterations, or renal dysfunction. The prevalence of MGUS may vary from 3 to 7% in the general population, especially after the fifth decade of life, and has been, in the past, related to chronic inflammatory or infectious diseases [3,4,5]. The issue has been has been usually dealt with by regularly monitoring through laboratory tests, often for decades, without any further consequence or evidence of a clinically relevant hematologic disorder.

More recently, the term “Monoclonal Gammopathies of Renal Significance” (MGRS) has been coined, appearing for the first time in 2012, in a report by the International Kidney and Monoclonal Gammopathy Research Group, to describe a renal abnormality or dysfunction initiated by deposition of a monoclonal (MC) immunoglobulin (Ig) component, even in the absence of multiple myeloma (MM) or any other clinically relevant lymphoproliferative disorder [6]. Actually, certain forms of MGUS without features of overt MM, previously known also as “smoldering myeloma”, fall into this newer category, since patients exhibit proteinuria or other signs of renal involvement. In MGRS, damage to the kidney could be massive, despite marginal clonal abnormalities of plasma cells at the bone marrow biopsy [6,7,8]. As an example, renal amyloidosis often originates from a non-myelomatous small clone releasing λ Ig light chains (LC). Glomerular deposition of amyloid substance results in a nephrotic syndrome (NS), with progressive renal failure, eventually leading to end-stage kidney disease [7,8,9]. At the same time, other organs, such as the heart and the liver, may be severely damaged by LC or amyloid deposition, resulting in fatal arrhythmias and/or organ failure. Acute kidney injury (AKI) is more often seen in patients with MM and massive accumulation of MC proteins deposited in glomeruli or renal tubules, obstructed by crystals or luminal “casts” [7,8]. Glomerulonephritis with immune complexes or complement deposits containing LC paraprotein have also been recently reported [7,8,9].

The present review, on the basis of a series of 24 consecutive renal biopsies in MGRS, deals with implications for renal function and prognosis, as well as the potential of tools, such as the renal biopsy, for assessing clinical risk and guiding the hematologist to the therapy of the underlying condition. 

## 2. Biology of Immunoglobulin LC and Significance of MC Components

**Figure 1:**
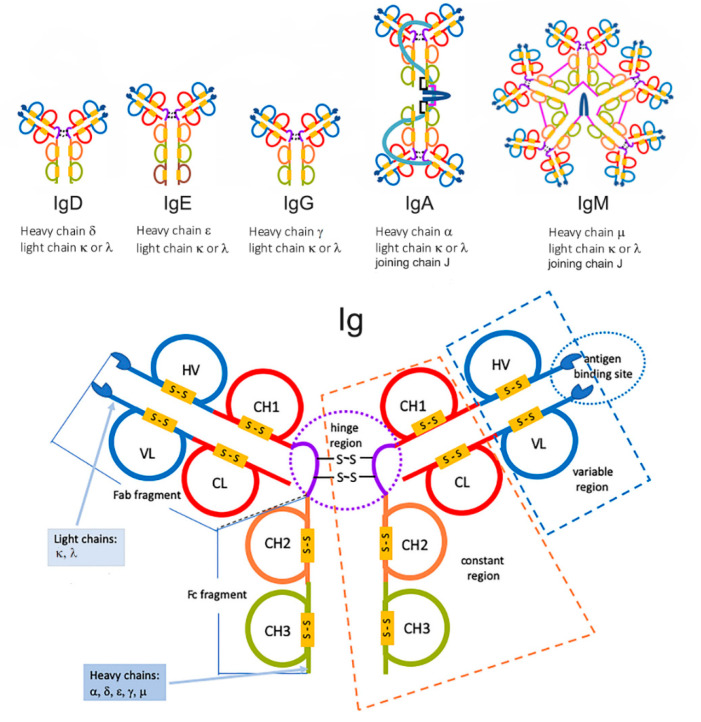
Basic structure of a human immunoglobulin. In the upper panels, the five isotypes, including dimeric IgA and pentameric IgM. See text for details.

Antibodies of all five Ig classes, namely A, D, E, G, and M, have a common four-polypeptide structure obtained by pairing two identical heavy chains (HC) with another couple of identical LC, joined together by interchain disulfide bonds, forming a Y-shaped molecule [10,11] (Figure 1). 

Paired disulfide bonds are located in a flexible “hinge” region, which creates two separate lobes and provides the structural flexibility needed to bind antigens of various shapes and surface. Circulating Ig usually forms dimers (IgG, IgA) or larger multiples, such as the pentameric IgM. Overall, the MW of a single IgG is about 150 kDa. HC and LC have an average MW of 50 and 25 kDa, respectively. Both HC and LC include variable and constant regions (domains) that are key to antibody function. Each domain contains 70–110 amino acids. Five types of HC, labeled α, δ, ε, γ, and μ, identify the five A-M classes of antibodies, which in turn differ in size and composition. The constant region of HC is identical in each Ig of the same isotype, but differs between isotypes. As an example, all IgA share the same sequence in their HC constant region, but these regions differ from those of IgD. The α, δ, and γ HC constant region is composed of three tandem Ig domains, whereas HC ε and μ contain four. The variable regions of the HC differ on the basis of the originator B-cells, so that each Ig “carries the signature” of the producing plasma cell [10,11].

Similar to HC, the two LC contained in a single Ig are identical to each other. Two types of these chains are present in mammals, labeled λ (lambda) and κ (kappa), with only one represented in each Ig. Each LC has one constant domain, followed by one variable domain, for a total length of approximately 215 amino acids. Igs can be, in turn, broken down into regions, with each region serving a different purpose. The Fab region (fragment antigen-binding region) is composed of one constant and one variable domain from each HC and LC. This part of the molecule is responsible for the classic Y-shape. The variable domain of the Fab region is also known as the Fv (fragment variable) region. Within Fab/Fv, “hypervariable regions” are positioned at one end of the variable domain, forming the beta-turn loops clustered next to each other in space. Clustering of the hypervariable loops at the tips of the variable regions where the antigen-binding site is located makes them suitable candidates for antigen recognition. The sequence heterogeneity of the three HC and three LC hypervariable loops creates virtually unlimited combinations, well suited to match the binding surface of the antigen. This antigen recognition sequence is often referred to as a complementarity-determining region (CDR) [10,11,12,13].

The remaining part of the Ab, namely the fragment crystallizable (Fc) region, does not contribute to binding the antigen, but is rather responsible for modulating the immune systems response to the formation of an antibody–antigen complex. The Fc region is composed of two HC constant regions that are isotype-specific. Glycosylation occurs at conserved positions in their Fc regions, making it critical to determine the rate of Ab clearance from the body. Once an Ab binds to an antigen, the Fc region binds to Fc receptors on leukocytes and resident tissue cells, to mediate a host of different physiological responses, including opsonization, degranulation of mast cells, release of cytokines and cytotoxic molecules, etc. This will eventually lead to the destruction of the pathogen. Depending on the class of the Ab, as dictated by the identity of the Fc region, its half-life and distribution throughout the body varies. Further, since Fc receptors are Ig isotype-specific, features of the immune response depend on the type of Fc region on the Ig, allowing for multiple immune response strategies against the same pathogen [9,10,11,12,13]. 

MGRS result from the deposition of assembled monoclonal Ig, as well as LC or HC fragments of variable size, released by plasma cells or plasmoblasts that undergo clonal proliferation, perhaps under the drive of helper T lymphocytes (or escaping the inhibitory influence of suppressor T cells) [8,9]. Despite descriptions of both LC and HC deposited within glomerular vessel walls, mesangial spaces and renal tubules (LC deposition disease, LCDD; HC deposition disease, HCDD), LC are 20-fold more frequently involved than HC fragments, most likely due to their limited size and MW, making transit across the filtration barrier easier and trapping within more likely [8,9,13]. At the same time, internalization of filtered LC by tubular epithelial cells via Fc receptors and the megalin/cubilin pathway is intuitively faster for polypeptides, with an average MW of 25 kDa, as opposed to 50 kDa of the HC. Another critical issue in determining filtration, deposition, and processing of the peptides is most likely the net electric charge. In cases of massive production and circulation of anionic paraproteins, there may actually be a rise of the serum anion gap in the context of metabolic acidosis, due to the contribution of such negative charges [14,15]. On the other hand, a strong anionic signature of circulating paraproteins may limit filtration and deposition within the similarly anionic microenvironment of the glomerular basement membrane and the podocyte slit membrane, whereas cationic paraproteins may be more permeant. Interestingly, IgGs are usually highly cationic, mostly belonging to the slow-migrating domain of the γ region of protein electrophoresis, while roughly one-half of IgAs are anionic and migrate into the α or β region, whereby they may contribute to an increase of the serum anion gap [14,15,16].

The toxicity of LC has been assessed in several in vivo and in vitro experimental settings. The paraprotein initially described in the urine of certain patients with renal disorders in 1847 by Henry Bence-Jones (Bence-Jones Protein, BJP) has been recognized as LC without the accompanying HC, since 1953, by Grabar and Williams [17]. BJP was later linked to the MC serum spike of MM by Edelman, who was awarded the Nobel Prize in 1962 [18,19]. LC can be accurately quantified by electrophoretic techniques, including immunofixation electrophoresis (IFE) [20]. Nowadays, BJP/LC have been better characterized in the urine by means of proteomic technologies, such as MALDI–TOF gas chromatography–mass liquid spectrometry (GC–MS), as low MW monomers, dimers, or high-MW polymers [21,22,23]. Conversely, BJP-type LC are present in serum as tetramers. A host of LC-accompanying proteins such as apolipoprotein E, vitronectin, and serum amyloid P (SAP) have been recognized in urinary exosomes (microvescicles obtained by sequential ultracentrifugation) in amyloidogenic plasma cell disorders [22,23]. The kidney metabolizes LC through sequential glomerular filtration, proximal renal tubular absorption, and local catabolism. BJP detected in the urine is evidently a spillover of LC that escaped reabsorption and metabolism by renal tubules. The injection of human BJP in experimental animals leads to deposition in renal tubules as protein casts, precipitates, or crystals; interestingly, λ LC from patients with amyloidosis are amyloidogenic when injected into the rat, as well [24,25]. More recently, Luciani et al. employed transgenic mice overexpressing Fanconi syndrome–associated κ LC, or primary cultures of mouse PT cells exposed to low doses of human κ LC, reporting that specific LC accumulate within lysosomes. They appear to alter lysosome dynamics and proteolytic function through defective acidification, thereby causing dedifferentiation and loss of the re-absorptive capacity of PT cells [25]. Ronco and coworkers found that λ LC isolated from patients with amyloidosis prompted a “macrophage” phenotypic transformation of cultured human mesangial cells, whereas a “myofibroblastic” phenotype was induced by LC isolated from subjects with deposition disease. Thus, taken together, these studies suggest that renal toxicity of MC proteins is specifically determined by the molecular species involved [26].

HCDD is a much rarer form of monoclonal Ig deposition disorder due to the synthesis of abnormal HC with C_H_1 and V_H_ region abnormalities, leading to their early secretion from plasma cells prior to conjugation with LC. Less than 50 cases have so far been reported in the literature, featuring isolated HC from IgA or IgD, mostly accumulated in the form of nodular glomerulosclerosis around mesangial deposits, similar to LCDD [27] (see below).

## 3. The Burden of Age: Epidemiology of MGRS

It is well-known that incidence and prevalence of MC gammopathies increase with age, so that the vast majority of cases is seen among subjects in their sixth decade of life or older [1,2,3,7,8]. In our own experience of MGRS (Table 1), patients had an average age of 62.5 ± 9.1 years (range 42–76) at the time of renal biopsy. 

Why this happens is unclear, particularly when compared to most other myelo- or lymphoproliferative disorders, which span across all age groups. One possible explanation is the high variability of Ab production in response to multiple antigens throughout a lifetime, giving rise to countless clones of plasma cells [9,10,11]. This increases the probability that over time a single clone escapes proliferative control. Moreover, the frequency of spontaneous mutations within the genome of plasma cells has been implicated; such a condition may provide an evolutionary advantage by offering a wide panel of preformed antibodies with epitopes potentially matching previously unknown antigens, without being actually primed by mechanisms involving antigen-presenting cells or T cells [28]. This could greatly enhance the speed of Ab-mediated adaptive immune responses to a previously unknown viral or bacterial challenge. On the other hand, genetic alterations occurring in the lymphoid germinal center prompt the normally non-dividing plasma cells to proliferate, eventually giving rise to MGUS [28]. One link between plasma cells’ dyscrasias, aging, and inflammation could also be represented by the known decrease in circulating B cells in aged individuals, since inflammation directly impacts on B lymphopoiesis [29]. Besides, there is a shift from naïve to memory Ig production, accompanied by impaired ability to form high-affinity protective Ab against newly encountered antigens. This result in expanded clones of B cells, often encountered in subjects with poor health status, along with a reduction of naïve B cells in the elderly, parallel to the expansion of memory B cells expressing a senescence-associated phenotype [29,30].

One implication of this phenomenon is a greater prevalence of plasma cell dyscrasias among the causes of AKI in aged patients, so that all events such as rapidly progressive (RP) renal failure in this age group should be thoroughly investigated by nephrologists for a possible MGRS. Tubular obstructive “cast” nephropathy, MC-associated glomerulonephritis, hypercalcemia from osteolytic lesions, or glomerular LCDD are examples of such possible causes of RP-ARF/AKI, which is discussed later. Above all, it should be kept in mind that many cases of MGRS are cryptic and can only be suspected if a serum electrophoresis and/or a search for a Bence-Jones proteinuria are performed. Immunofixation of serum globulins would be the next step, in order to identify an MC subset of γ or β globulins and an imbalance of LC from the standard κ/λ ratio of 0.25–1.65 (0.37–3.1 if renal failure stages 4–5 K/DIGO).

## 4. Directions: Hematologist to Nephrologist or Vice Versa?

An interesting issue concerning MGUS and MGRS relates to the professional collaboration between hematologists and nephrologists. One would expect that all patients referred primarily to the hematologist because of a plasma cell dyscrasia would be seen by the nephrologist whenever an alteration of urinalysis or a previously unobserved reduction of renal function is detected during a laboratory workup. This is certainly so, with the notable exception of individuals who escape early identification, because serum electrophoresis is seldom requested during routine follow-up by general practitioners. In Table 1, we report our own renal biopsy experience in individuals who came to our attention in the Renal Outpatient Clinic because of persistent urinary abnormalities. All of them had proteinuria, 20 out of 24 in the nephrotic range, 5.7 g/day ± 2.9 as a whole, whereas age-adjusted eGFR was reduced in 14 out of 24 patients. Subsequent workup identified in all of them an MC serum and/or urine component. These cases were not referred to us by hematologists, and just one-third (33.3%) had a subsequent histologic and clinical diagnosis of MM. This indicates that, in nearly 80% of the individuals in which we detected an MGRS by renal biopsy, the major clinically relevant sign of a plasma cell dyscrasia was a renal abnormality, mostly proteinuria, with a recent reduction of eGFR in 58.3%. A bone-marrow histology would have not granted clone-directed therapy in most cases. Interestingly, 13 out of 24, or 54.1%, were AL renal amyloidosis, while 37.5% were diagnosed as LC deposition disease. Thus, whenever we performed a renal biopsy in a patient with an MC disorder and a clinically proven MGRS (proteinuria > 1 g/dl of recent onset, progressive or rapid decrease of renal function, tubular dysfunction in the absence of other Durie–Salmon criteria for multiple myeloma, etc.), the findings were consistent with MC protein deposits. All patients entered afterward in a hematology follow-up program, undergoing treatment with various drug regimens, mostly including proteasome inhibitors, with autogenic stem cell transplantation in three cases. 

Of course, our observation does not rule out referral from the hematologist to the renal clinic, but suggests that a patient with histologically proven MM undergoing treatment generally does not require a renal biopsy, unless there is uncertainty concerning whether or not to treat the plasma cell disorder. Most frequently, the request for a nephrology consultation arises in such cases from an abrupt impairment of renal function. This type of AKI is usually related to tubular obstruction by large amounts of filtered LC, a histological feature known as “cast nephropathy” [31,32]. A renal biopsy is useful to confirm this suspicion and guide rescue therapy. As cast nephropathy is also seen in the NS, a setting in which tubular obstruction by proteins is one of the recognized causes of AKI, the distinguishing feature of MGRS is a strong positivity of the immunofluorescence for κ or λ chains within the tubular lumina [6,8,31,32]. Since LC in a normal κ/λ ratio of approximately 1:1 are frequently found in the biopsies of patients with the NS, resulting from tubular injury by filtered serum proteins, the pathologist should attribute obstruction to an MGRS if just one subclass of LC is represented, either κ or λ. In such cases, we and others set out to support clone-oriented hematologic therapy with apheretic techniques, such as plasmapheresis, high-cutoff membrane hemodialysis, or hemofiltration with endogenous reinfusion, in order to rapidly remove circulating LC [33,34]. Another circumstance that may require support from a renal biopsy is the search for renal amyloidosis [7,8,9,35], whenever no other evidence could be obtained by Congo Red staining of a bone marrow biopsy, abdominal fat, or minor salivary glands tissue. The interest in such differential diagnosis, i.e., MM vs. renal amyloidosis, arises from the different pharmacologic approaches that could be undertaken [35].

Along these lines, attention should be paid to a relatively rare form of MGRS that can escape observation due to the absence of an MC component in the serum electrophoresis. Such is the case of non-secretory, “micromolecular” myeloma, which could be suspected by an experienced observer whenever the serum levels of γ-globulins are exceedingly low, even for a patient with the NS [36,37]. In this clinical presentation, a γ-globulin fraction < 8% at the serum electrophoresis (about 5.5 g/L or less when the patient is not nephrotic) is an indicator that overall production of γ-globulins is depressed. This is common in the NS due to urinary losses, but should never occur in an otherwise immunologically normal individual. Bone-marrow infiltration and substitution by a non-secretory clone of plasma cells should then be suspected, which may have depressed the polyclonal synthesis of other Igs [36,37].

## 5. Renal Biopsy in Persistent Urinary Abnormalities

Renal biopsy could provide an outstanding contribution to the early and accurate diagnosis of an MGRS. Its utility goes beyond the actual identification of the renal lesion that underlies persistent urinary abnormalities or AKI. Evidence of ongoing damage to the kidney by deposition of an MC protein, glomerulonephritis with LC deposition, or infiltration by lymphoma cells releasing a monoclonal LC may prompt a more aggressive diagnostic approach, including a serum-free LC assay (FLC), serum and urine immunofixation, bone marrow or lymph node biopsy, total body CT or CT-PET scan, skeletal Rx, echocardiography for amyloid deposition, etc. Hence, an MGUS, which is usually handled by a “watchful wait” approach, may be upgraded to a systemic disorder with impact on distant organs, thus deserving treatment, even if it does not fulfill the criteria for a diagnosis of MM or other major hematological disease. On the other hand, the search for reliable serologic or urinary markers of ongoing renal damage has not yet provided a clear, practical clinical approach. As an example, proteomic GC–MS studies on exosomes, microvescicles extracted from the urine by ultracentrifugation, are promising and could be very informative on the nature of the paraproteins excreted or retained by the kidney [21,22,23]. However, their diagnostic application is still lagging behind conventional tests, such as protein electrophoresis, immunofixation, or FLC. The renal biopsy is, on the other hand, reasonably accessible, less invasive than a myocardial or liver biopsy, and provides in well-equipped laboratories a wealth of information.

**Figure 2:**
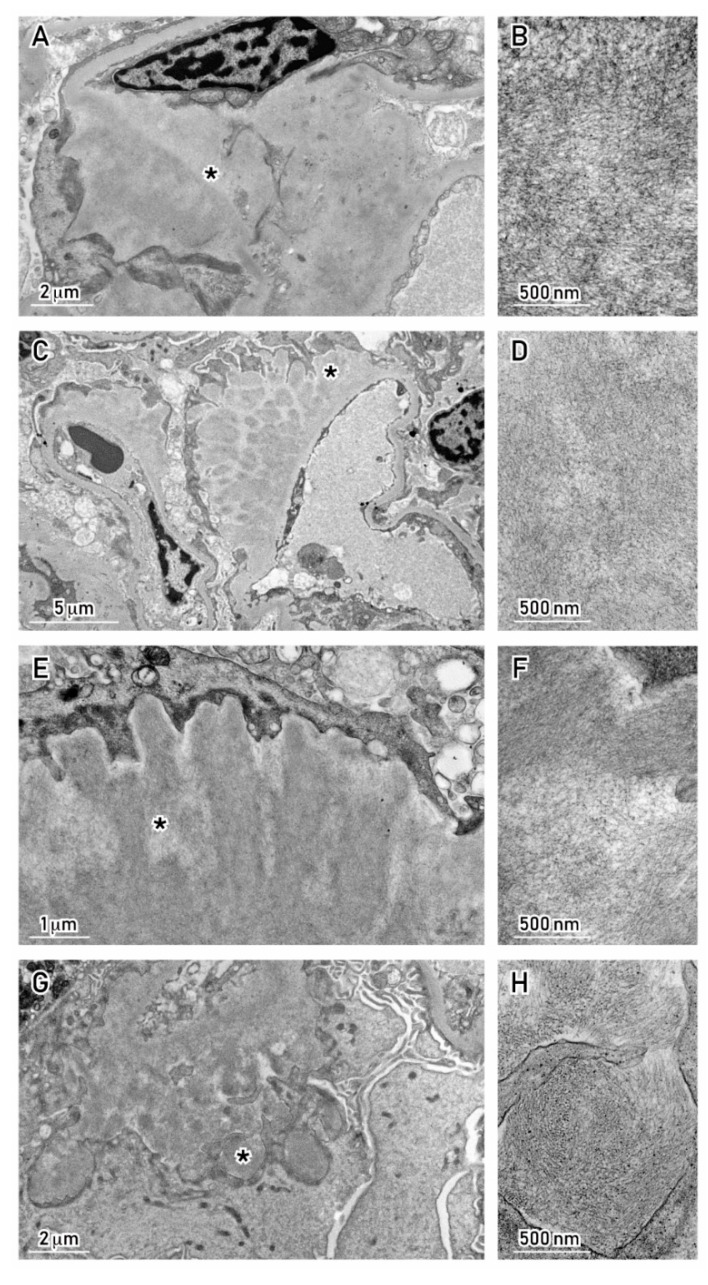
(**A,B**) AL Amyloidosis, IgG λ. Amyloid deposits in the mesangium with haphazardly arranged fibrils, ∅ ÷8.5 nm. (**C,D**) AL Amyloidosis, IgA λ. Amyloid deposits along the capillary loops with haphazardly arranged fibrils, ∅ ÷7.6 nm. (**E,F**) AL Amyloidosis, IgA λ. Bundles of amyloid fibrils forming “haystacks” perpendicular to the GBM (same case as in (C,D)). (**G,H**) AL Amyloidosis, IgG κ in B-cell lymphoma. Amyloid deposits causing podocyte foot process effacement and widening of basement membranes with randomly arranged and nonbranching fibrils, ∅ 8 nm. Transmission Electron Microscopy, uranyl acetate; Morgagni 268D TEM–FEI, Hillsboro, OR, USA. Abbreviations: GMB, glomerular basement membrane; MM, multiple myeloma. The fibrillar pattern of this area (*) is shown magnified in the micrograph(s) on the right side.

In order for a renal biopsy to be properly diagnostic, some standards must be met in addition with ordinary light microscopy with hematoxylin/eosin, PAS, silver methenamine, and Masson’s trichrome [38,39,40,41]. Accurate immunofluorescence screening for κ and λ LC, as well as possibly HC, should always be included. If there is any doubt on the relative fluorescence intensity of LC (which should match the κ/λ LC ratio in the serum FLC or immunofixation assays), immunoperoxidase could also be used, employing sensitive antisera. Sections should also be examined under polarized light microscopy, following Congo Red or Thioflavin T staining. Since these stains are technically delicate and operator-dependent, a negative report should always be confirmed by electron microscopy (EM) [41]. This technique is the “gold standard” for (immune) deposit disorders of the kidney, and in this group of diseases, identification of the typical 7.5–10 nm random-oriented fibrils is highly sensitive and specific for amyloidosis [39,40,41,42,43,44,45] (Figure 2A–H). 

**Figure 3:**
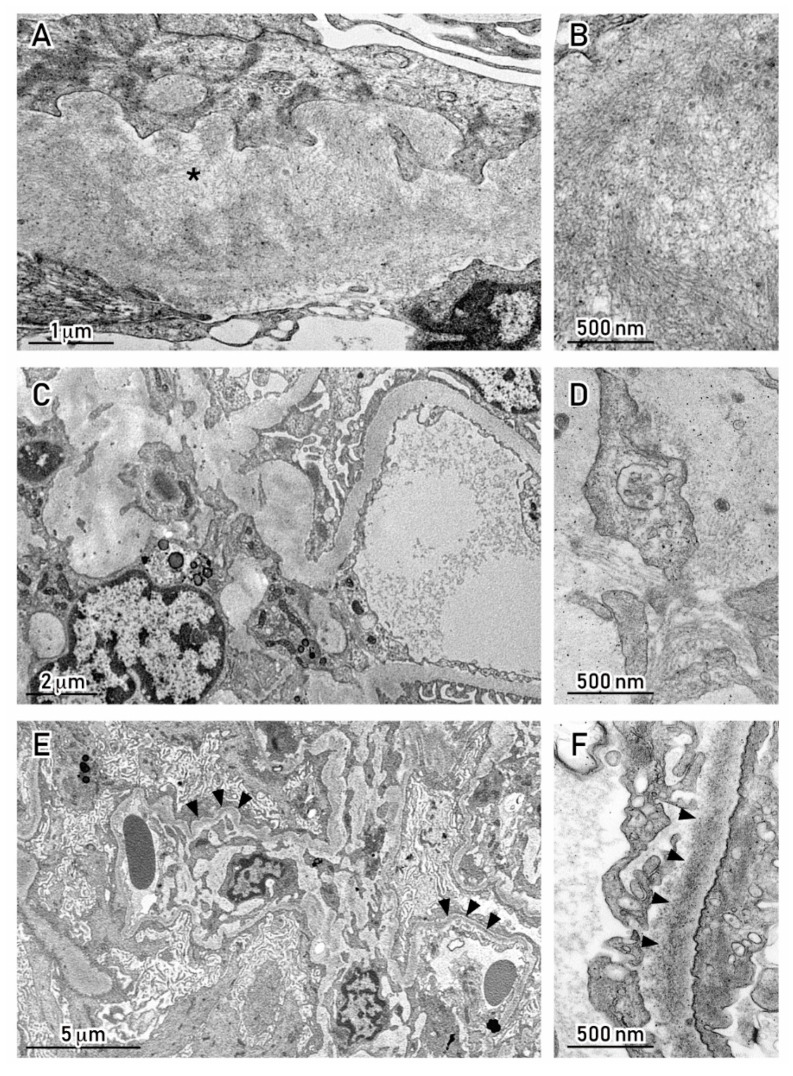
(**A,B**) LCDD, IgG λ. Fibrillary deposits in the subepithelial region of capillary wall, with straight fibrils over a lucent background, ∅ ÷12.5 nm. Congo Red stain was negative. (**C,D**) Cast nephropathy, IgG κ MM. Scant deposits in the mesangial matrix with fibrils also organized in parallel arrays, ∅ ÷15 nm. (**E,F**) LCDD, IgG κ. Band-like deposition of electron-dense granular material running along the GBM (black arrowheads). Transmission Electron Microscopy, uranyl acetate; Morgagni 268D TEM–FEI, Hillsboro, OR, USA. Abbreviations: GMB, glomerular basement membrane; LCDD, light chain deposition disease; MM, multiple myeloma; The fibrillar pattern of this area (*) is shown magnified in the micrograph(s) on the right side.

Amyloid subtyping could be further carried out by GC–MS, as mentioned earlier, with the goal of distinguishing inflammatory amyloid (AA) from AH, LC-related amyloidosis [22,23]. In addition to amyloidosis, other forms of MC protein deposition have been described based on EM features in these patients, such as fibrillary GN (Figure 3A–D), cryoglobulinemic GN, or immunotactoid glomerulopathy [7,44,45]. 

These nephropathies are characterized by a mesangial or mesangio-proliferative pattern on light microscopy, but deposits are not in the form of amorphous immune complexes, as seen in glomerulonephritis. They are typically Congo Red–negative, while the EM appearance is either as randomly oriented fibrils with a thickness of 13–29 nm, or as orderly bundles of microtubules with an average diameter in the range of 20–30 nm (cryoglobulins) to 10–90 nm (immunotactoid) [46]. More than typical features of MM, fibrillar and cryoglobulin/immunotactoid GN are encountered in lymphoproliferative disorders, such as B-cell lymphomas [7,41,42,44].

EM is also useful to locate immune deposits along the glomerular basement membrane (GBM) or in the mesangium, as may be seen in GN with MC protein deposition (see below) (Figure 3E,F) [42,44,45].

## 6. Mechanisms by Which MC Proteins Damage the Kidney

There are two major mechanisms by which MC proteins or derivatives, such as amyloid substance, induce kidney injury. First and foremost, physical accumulation of filtered proteins at the level of the filtration barrier (GBM, mesangial cells and related endothelial and visceral epithelial layers of the capillary wall) or within renal tubules [7,8,9,40,41,42]. Occlusion of capillary or tubular lumina is probably not the major reason for a sharp decline of renal function, often falling into the category of “rapidly progressive renal failure”. Actually, protein casts have been found in renal biopsies or autopsy samples of >60% of patents with MM [38]. However, many such patients do not show signs of AKI or rapidly progressive renal failure until very advanced stages of the disease and in the presence of severe dehydration. On the other hand, the key issue is probably local toxicity of LC and amyloid fibrils to glomeruli and tubules (see next paragraph). As mentioned earlier, filtration of circulating LC, or rarely HC, occurs through a balance of all the physical forces involved, that is, transmembrane hydraulic pressure gradients, permselectivity of the GBM (“pores”), oncotic forces across the GBM, and net electric charge. The molecular size and radius of the LC make transit across the GBM and podocyte slit diaphragm possible even when there are no physical breaks across the filtration barrier. For comparison, albumin has a MW of 69 kDa, with a molecular radius of 3.55 nm, resulting in a relative concentration in the ultrafiltrate of <0.01 (filtration fraction < 1% of circulating serum albumin). LC instead have MW of 10–25 kDa, so that filtration may occur easily on a pure physical basis (pore size of the GBM). However, the net anionic charge of a normal GBM (mostly related to the abundant heparansulfate and glycosaminoglycans content) is likely to act as an electrostatic barrier for negatively charged LC (see Section 2, on biology of Ig LC).

The role of inflammation is controversial, since amyloid material or LC deposits usually do not elicit a major chemotactic response, thus making leukocyte-mediated endothelial damage or tubulointerstitial inflammation unlikely [39,41,42]. Occasionally, however, cases of acute interstitial nephritis with extensive lymphocyte and plasma cell infiltration have been reported, clearly unrelated to the presence of tubular casts or LC crystal precipitation [41,42]. On the other hand, tubular basement membrane ruptures in LCDD or cast nephropathy may appear surrounded by macrophages, occasionally forming granulomatous lesions. GNF with MC protein deposition is rarely characterized by leukocyte infiltration, although cellular crescents have been noted in selected cases with an extracapillary proliferative pattern.

## 7. How to Assess the Link between MC Proteins and Renal Injury: The Role of the Renal Biopsy

Damage to the kidney by deposited LC and, more rarely, HC alone or in combination with LC is testified by abnormalities of urinalysis, proteinuria, signs of tubular injury, and/or a reduction of GFR with variable speed of progression [47]. The renal biopsy is often quite informative about the causes of the functional impairment and where critical damage is actually occurring [8] (Table 2). 

A renal biopsy should always be obtained and examined by an experienced pathologist whenever an MGRS is suspected [41,42,44,45,47]. For example, nodular deposits of LC or HC within the capillary walls and the mesangium, that resemble nodular diabetic glomerulosclerosis (Kimmelstiel–Wilson glomerulopathy), indicate a predominant involvement of glomeruli, the so-called glomerular LCDD. This translates into heavy proteinuria progressing to the NS, along with early loss of function that may even occur within few weeks or months, with features of rapidly progressive AKI [7,8,9]. These deposits are usually faintly PAS-positive, unlike the brisk staining of diabetic glomerulopathy. Diagnosis is based on immunofluorescence, which shows the selective κ or λ LC nature of the deposits [41,44]. Rare forms of crystalline glomerulopathies have been described, mostly involving podocytes and parietal glomerular epithelial cells, along with proximal tubular deposition [47,48]. These aggregates of LC are thought to occur because of a unique resistance of certain paraproteins to proteolysis within lysosomes [47,48,49,50]. Related to these crystalline deposits is a rare deposition of “crystalglobulins” within the glomerular capillary walls; these deposits may also involve the skin microcirculation, with ulcers and purpuric lesions [51].

Tubulointerstitial LCDD is even more common [52,53,54]. Early clinical signs of tubular involvement can be grouped into the acquired Fanconi syndrome, i.e., altered acidification mechanisms with tubular acidosis, tubular proteinuria (non-selective, mild, usually < 1.5 g/day), glycosuria, aminoaciduria, low serum phosphate, and uric acid due to impaired tubular reabsorption [55]. Selective defects can also be seen, based on the extent of tubular damage.

Structural changes are quite apparent in the renal biopsy, beyond the obvious positivity of IF for LC or rarely HC. Tubules appear enlarged, with thickened and tortuous basement membranes, lined by deposits on the outer side. Cells undergo various processes of apoptosis, “blebbing”, and vacuolization, with loss of the brush border in the proximal tubule. In advanced stages, detachment from the parietal monolayer can be noted, along with clumps of cellular debris within the lumen, often engorged and repleted with amorphous proteinaceous material, i.e., precipitated LC [38,41]. The clinical features can progress to an obstructive tubulopathy known as “cast nephropathy”, whereby LC precipitates are mixed with sloughing tubular cells and the typical debris of acute tubular necrosis (ATN) [8,38,41,56,57]. This entity could be suspected whenever the patient with an MC serum component has shown earlier signs of tubular dysfunction, and suddenly develops AKI with a sharp reduction of urine output. Aggressive diuretic therapy or dehydration from any cause may trigger this complication, with often requires urgent hemodialysis, possibly coupled with paraprotein apheretic techniques [33,34].

Glomerulonephritides with MC Ig deposits, also known as “Proliferative GN with Monoclonal Immune Deposits (PGNMID)”, have been only recently described, usually as membrano-proliferative GN with IgG3 selective deposition (no immune complexes), with or without an MC κ chain [7,58,59] (Table 2). Deposits have a granular appearance on EM, easily distinguished from cryoglobulins or fibrils, while structural changes involve mostly capillary endothelial cells and mesangial cells, which increase in number and accumulate extracellular matrix, as well as electron-dense, deposits. Another type of PGNMID that has been associated with MC gammopathies is “dominant C3” GN, a proliferative lesion of glomeruli resulting from activation of the alternative complement pathway [60,61,62]. Actually, C3GN is part of a triad of diseases characterized by uncontrolled activation of the alternative pathway, also including type 2 membranoproliferative GN (“dense deposit disease”, DDD), and the hemolytic–uremic syndrome (HUS). All of these forms are reportedly linked to genetic or acquired defects of complement regulatory proteins, such as factor H (CFH), factor I (CFI), membrane cofactor protein (MCP), decay activating factor (DAF), or thrombomodulin [60,61,62,63]. The observation that λ LC or MC Ig can bind to and impair the function of CHF has led to the hypothesis that the alternative pathway can be activated by circulating MC proteins during MGRS or lymphoproliferative disorders [60,61,62,63]. Another possibility is that the presence of MC LC could obscure IgG within immune complexes, leading to the detection of a dominant C3 immunofluorescence pattern. It has been shown that pronase digestion on paraffin-embedded sections might reconcile at least certain cases with a classic pattern of IgG-containing immune complexes [7,62]. The same technical issue has been raised by Larsen et al. for biopsies showing large subepithelial C3-dominant deposits in a membranous pattern, which upon pronase digestion of paraffin-embedded sections turned out a brisk IgGκ positivity [64].

## 8. When and How to Treat MGRS

The answer to this question is rather controversial. For decades, hematologists have been following MGUS closely, usually without opting to treat unless systemic signs prompted to do so. The key indicators of a switch from MGUS to MGRS or MM—and thus the need for treatment—are evidence of bone marrow infiltration by MC plasma cells or precursors, a serious imbalance in the κ/λ serum ratio, or deposition of amyloid at one or more of the classic sites (bone marrow biopsy, kidney, heart, liver, subcutaneous abdominal fat, “minor” salivary glands, rectal mucosa, etc.). In amyloidosis, a quantitatively relevant MM clone may be absent, yet the amyloidogenic clone needs to be treated aggressively to prevent widespread organ damage [35,65]. In recent years, development of the MGRS concept has brought attention to the kidney as a major target of circulating “nephrotoxic” MC proteins, along with the agreement that all efforts should be undertaken to preserve renal function [6,7,13,66]. This is also critical in view of the growing indication to bone-marrow auto- or allogenic transplantation to treat MM and related plasma cell dyscrasias [67,68,69,70,71].

Thus, there is a consensus toward a clone-directed approach whenever an MGRS results in impaired glomerular or tubular function, and a renal biopsy confirms the deposition/localization of an MC protein within the kidney, along with evidence of an imbalance of the serum κ/λ ratio or the FLC profile [65,66,67,68,69,70,71]. Evidence of LCDD or PGNMID is an example of such a situation. Table 3 lists the major lines of treatment currently employed for MGRS, closely matching the schemes applied to MM and/or amyloidosis [67,68,69,70,71].

Of course, the first sign of impending damage to the kidney is a rapid reduction of GFR, not otherwise explained by frequent confounding factors in aging individuals, as most subjects with MGRS indeed are. Therefore, ischemic kidney disease, diabetes, and cardiac failure should be ruled out. Medications that could impact on RBF and GFR should be withdrawn, such as ACE-inhibitors, ANG II-receptor antagonists, NSAIDs, etc. Dehydration due to concurrent conditions (gastroenteritis, excess diuretics, malnutrition, and hypercalcemia) should be corrected. If no clinical explanation can be found, and/or urinalysis shows proteinuria or hematuria, a renal biopsy should be obtained to guide therapy.

## 9. Conclusions

In summary, MGRS are a challenge for both hematologists and nephrologists, and they are one of the grounds where collaboration is most profitable. Early diagnosis is key to spare end-organ damage by LC or amyloid fibrils, not only to the kidney itself, but also to the heart, liver, and the vascular compartment at large. A thorough evaluation of renal function, followed by a renal biopsy, should be recommended as a standard approach to MGRS, parallel to, or even preliminary to, the hematology workup. Notably, most such patients are seen initially due to renal abnormalities, and evidence of an MC component comes as an unexpected finding during initial laboratory evaluation. The availability of renal replacement/apheretic techniques may later bridge the gap between recognition of kidney injury and effective response to pharmacologic treatment. Clone-directed therapies and avoidance of all medications that may negatively impact on renal function could greatly benefit patients with MGRS, whose overall prognosis may be more favorable than in overt MM, provided that renal or cardiac failure do not set in, at least early in the course of disease. Therapeutic options with new molecular tools or stem cell transplantation take obviously great advantage by preservation of the integrity of renal function [69,70,71].

## Figures and Tables

**Table 1:** Renal histology in 24 consecutive patients with monoclonal gammopathies of renal.

Significance (MGRS)					
Patient	Age	M/F	eGFR, mL/min	uProt g/day	Diagnosis
T.A.	64	M	52.2	11.0	AL Amyloidosis, IgA λ mMM
M.M.B.	72	F	10.6	5.0	AL Amyloidosis, IgG λ
L.B.N.	51	F	36.6	1.9	AL Amyloidosis, IgG λ mMM
G.C.	65	M	33.1	5.2	LCDD, IgM λ
G.C.	54	M	108.5	5.0	AL Amyloidosis + FibGNF, IgM λ
R.C.	68	M	100.0	4.8	AL Amyloidosis, IgA λ
E.D.B.	72	M	30.4	1.1	LCDD, IgG λ
R.D.C.	72	F	9.5	7.5	AL Amyloidosis, IgG λ
I.D.T.	71	F	50.7	13.2	LCDD, IgG λ
L.F.	66	F	25.1	8.8	LCDD, IgG κ
R.G.	54	F	160.2	3.1	AL Amyloidosis, IgA λ MM
G.G.	60	F	32.3	4.1	LCDD, IgG κ
M.L.	59	M	94.0	5.3	AL Amyloidosis, IgG λ
E.L.C.	70	M	84.3	4.6	AL Amyl., B-cell lymphoma, IgG κ
M.L.	45	M	96.7	8.8	LCDD, IgG κ
P.L.	70	F	90.1	5.2	AL Amyloidosis, IgG λ
B.M.	43	F	11.9	5.0	Cast nephropathy, IgG κ MM
G.M.M.	42	F	107.1	2.2	LCDD, IgG κ
E.P.	54	F	120.5	4.4	AL Amyloidosis, IgG λ
A.P.	76	M	12.4	10.0	Cast nephropathy, AL Amyl., IgG λ
F.P.	66	M	39.4	7.2	AL Amyloidosis, IgG λ MM
M.C.S.	52	F	65.3	5.8	LCDD, IgG κ MM
D.S.	64	M	45.8	8.5	LCDD, IgG κ MM
V.T.	62	M	26.3	1.2	AL Amyloidosis, IgA λ
Data are expressed as mean ± SD	62.58 ± 9.16		60.00 ± 40.54	5.73 ± 2.94	

Abbreviations: eGFR: estimated glomerular filtration rate (CKD-EPI equation); uProt: proteinuria; LCDD: light chain deposition disease; MM: multiple myeloma; mMM: non-secretory MM; FibGNF: fibrillary glomerulonephritis.

**Table 2:** Major known clinical/pathological presentations of monoclonal gammopathies/MGRS of Renal Significance (MGRS).

Major Known Clinical/Pathological Presentations
Light chain deposition disease (LCDD, glomerular/tubular)
Heavy chain deposition disease (HCDD)
Tubulointerstitial LCDD with Fanconi syndrome
Tubular obstructive “cast nephropathy”
AL amyloidosis
Proliferative glomerulonephritis with monoclonal immune deposits (PGNMID)
“Dominant C3” glomerulonephritis
Fibrillary glomerulonephritis
Immunotactoid glomerulonephritis
Cryoglobulinemic membranoproliferative glomerulonephritis (“non-infectious”)
Nephritis with crystalline inclusions (“crystalline podocytopathy, tubulopathy”)

**Table 3:** Current major clone-directed therapeutic strategies for MGRS/MM.

Major Regimens/Associations
Melphalan + prednisone (MP) ± thalidomide (MPT) *
Thalidomide + dexamethasone (TD)
Lenalidomide + dexamethasone (RD)
Bortezomib + dexamethasone (VD)
Bortezomib + melphalan + prednisone (VMP)
Bortezomib + thalidomide + dexamethasone (VTD)
Bortezomib + cyclophosphamide + dexamethasone (CyBorD, VCD) *
Bortezomib + lenalidomide + dexamethasone (VRD)
Carfilzomib + cyclophosphamide + dexamethasone (CCyD) *
Carfilzomib + lenalidomide + dexamethasone (KRD)
Pomalidomide + dexamethasone (Pom/Dex)
Carfilzomib + pomalidomide + dexamethasone (KPD)
Autologous Stem Cell Transplantation (ASCT) *
CD38-targeting immunotherapy: monoclonal Abs Daratumumab *
Daratumumab *
Elotuzumab
Anthracyclines, tetracyclines
Doxorubicin *
Doxycicline *
Aggresome inhibitors
Panobinostat

* Note: often employed or particularly suited for the treatment of AL amyloidosis.
